# Factors associated with medication adherence among hypertensive patients in Namibia

**DOI:** 10.4102/hsag.v31i0.3178

**Published:** 2026-03-13

**Authors:** Daniel O. Ashipala, Medusalem H. Joel, Abraham V. Nghikevali, Anton Victorinu

**Affiliations:** 1 Department of General Nursing Sciences, Faculty of Health Sciences and Veterinary Medicine, University of Namibia, Rundu, Namibia; 2 Department of General Nursing Sciences, Faculty of Health Sciences and Veterinary Medicine, University of Namibia, Windhoek, Namibia

**Keywords:** factors, medication adherence, hypertensive medications, patients, hypertension

## Abstract

**Background:**

Hypertension is a major public health concern, with non-adherence to treatment undermining disease management and control. In Namibia, little research exists on medication adherence among hypertensive patients, particularly in the Kavango East region, Namibia.

**Aim:**

This study examined demographic, clinical, behavioural, and health system factors associated with medication adherence among hypertensive patients at a regional hospital in the Kavango East region, Namibia.

**Setting:**

The study was conducted at the outpatient department of a regional hospital in north-eastern Namibia.

**Methods:**

A quantitative, cross-sectional approach was used with 200 hypertensive patients selected through consecutive sampling. Data were collected by using a self-administered questionnaire and analysed with Chi-squared tests and logistic regression to assess associations with adherence.

**Results:**

Non-adherence to antihypertensive medications was common, with 62% (*n* = 124) reporting missed doses. Key reasons included forgetfulness (*n* = 112; 56%), side effects (*n* = 88; 44%), financial constraints (*n* = 74; 37%), transport barriers (*n* = 64; 32%), and poor knowledge (*n* = 52; 26%). Significant predictors of poor adherence included forgetfulness (adjusted odds ratio [AOR] = 0.39, *p* = 0.006), side effects (AOR = 0.42, *p* = 0.011), financial constraints (AOR = 0.40, *p* = 0.009), and transport difficulties (AOR = 0.44, *p* = 0.019). Employment (AOR = 0.52, *p* = 0.041) and living with hypertension for ≥ 5 years (AOR = 1.75, *p* = 0.047) were protective, while men were 1.48 times more likely to adhere than women.

**Conclusion:**

Adherence to antihypertensive therapy is undermined by socioeconomic, behavioural, and health system barriers.

**Contribution:**

This study provides much-needed evidence on the determinants of medication adherence in Namibia. It offers guidance for public health strategies, community awareness, and improved hypertension management outcomes.

## Introduction

Hypertension is one of the primary contributors to death globally and is largely associated with coronary heart disease. Globally, it has been noted that hypertension causes 1.332 million deaths yearly (Wu et al. 2025). In addition, it is also estimated that hypertension will be prevalent in 1.56 billion adults by 2025 and that awareness about hypertension, its prevention, and treatment is particularly limited in low- and middle-income countries (World Health Organization [WHO] 2023). Indeed, the Global Burden of Disease Study found that hypertensive heart disease accounted for 17.5 million disability-adjusted lives (Liu et al. 2024). Optimal blood pressure (BP) control is key to preventing hypertension-related complications and deaths, while taking antihypertensive medications can also effectively lower hypertension-related complications, thus improving survival rates (Wang et al. 2024).

High BP does not typically cause obvious symptoms, which is why people often call hypertension the ‘silent killer’ (Omar et al. 2020). Yet, high BP that persists for a long time is a major risk factor for strokes, heart disease, vision loss, coronary artery disease, atrial fibrillation, peripheral arterial disease, chronic kidney disease, and even dementia (Abraham et al. 2024). Medication adherence is the extent to which a patient takes their medication, based on agreed-upon recommendations or prescriptions (Vrijens et al. 2017). It is imperative that patients who receive medications take their medication according to how it is prescribed, as this is critical to controlling BP. Several studies have shown that adherence to antihypertensive drug treatment does result in better BP control and reduced risk of cardiovascular disease (Herenda, Jusufović & Musanović 2025). Patients who fail to adhere to their antihypertensive treatment have a higher risk of hospitalisation or other adverse effects (Stolfo et al. 2025).

Cardiovascular disease (CVD) remains a severe health problem in lower- and middle-income countries (LMICs), including Namibia. Globally in 2008, approximately over 20.5 million died from CVD, of which more than half of the CVD-related deaths were because of hypertension (Raj, Garg & Kaur 2025). According to Hinneh et al. (2024), about 32% – 57% of adults in sub-Saharan Africa are suffering from hypertension. Namibia is one of the countries in sub-Saharan Africa with the highest hypertension prevalence (Nakwafila et al. 2022). Despite this profile, little is known about the potential ways in which adherence to antihypertensive therapy can be improved in Namibia.

In Namibia, the prevalence of hypertension among women and men aged 35–64 years has skyrocketed and is now ranging from 44% to 57%, which is significantly higher than the global (18%) and African (27%) prevalence, respectively (Nakwafila et al. 2022). On the contrary, the LMICs have in the past years witnessed a gradual increase in the number of people with hypertension, yet one in three people know of their hypertension status, whereas 8% of people have controlled BP (Schutte et al. 2021). However, only 33% – 66% of hypertensive patients in LMICs are currently receiving antihypertensive medicines (Mishra et al. 2025). The prevalence and potential high mortality rate demand that healthcare systems, including primary healthcare facilities in LMICs, are strengthened. These interventions are necessary to prevent and control hypertension and sustainably improve health outcomes. This move will also help the United Nations to achieve its Sustainable Development Goal 3.4 of reducing premature mortality from non-communicable diseases by one-third by 2030 (United Nations 2025). This goal includes strategies to optimise adherence to antihypertensive therapy and enhance access to affordable medicines to treat non-communicable diseases, including hypertension, by 80% (Pickersgill et al. 2022).

To address this already considerable and still growing public health problem, the primary healthcare centres guided by the national Primary Healthcare Policy now provide free universal access to essential antihypertensive medicines and other aspects of care (Fanda et al. 2024). Namibian primary healthcare facilities are strategically located among underprivileged communities and have a critical role in providing hypertensive Namibians with access to care (Nashilongo et al. 2017). According to Mishra et al. (2025), easy and free universal access to antihypertensive medicines and care also removes the financial barrier, which is often an issue in LMICs.

Literature suggests that nearly half of the known hypertensive sufferers in low- and middle-income countries do not take medication, and more than half of those treated still have uncontrolled BP (Schutte et al. 2021). Factors relating to the patients themselves, physicians, and the healthcare system may be responsible for this poor level of treatment and suboptimal control of hypertension, but few studies have been conducted in LMICs to assess and describe these factors (Byiringiro 2023). This study sought to answer the following research questions at a regional hospital in the Kavango East region, Namibia: what demographic, clinical, behavioural, and health system factors are associated with medication adherence among hypertensive patients?

## Research methods and design

### Study design

A quantitative cross-sectional study was conducted among hypertensive patients to identify the factors associated with treatment adherence among hypertensive patients and their associated predictive factors in a state hospital in Kavango East Region, Namibia. Quantitative research is a systematic, objective process that uses numeric data obtained from a subgroup in order to generalise the findings to the population being studied (Maree 2016). This approach uses randomised samples, allowing one to reach a higher sample size, and one can collect information quickly when using this approach (Bhandari 2020). Hence, the approach was used to quantify the factors associated with non-adherence to antihypertensive medication at a state hospital in Kavango East Region, Namibia.

### Study setting

The study was conducted at the outpatient department of a selected regional hospital located in the northeast of Namibia between March 2022 and April 2022. This government hospital has 300 beds and caters to hospital and clinic referrals from the Kavango East, Kavango West, and Zambezi regions. According to the Outpatient Department Monthly Summary Report form of 2021, 401 patients were treated for hypertension (new cases and follow-up).

### Population

The researcher used the population of 401 patients who were treated for hypertension as informed by the Outpatient Department Monthly Summary Report form of 2021. The sample size of this study was determined by using a standard formula; sample size = N/(1 + Nxa ^ 2) for a cross-sectional study (De Vos et al. 2017), where:

N = sample size

N = total population

A = total confidence limit at 5%, which is 0.05.

The calculation will be performed according to Sekaran and Bougie (2016) as below:

N = N/1 + N x a ^ 2

N = is the sample size

N = is the total population (which is 401)

A = is the total confidence, which is 0.05

N = 401/1 + 401 x (0.05) ^2

N = 401/2.0025

N = 200.

Hence, the sample size for this population was 200.

### Sampling strategy

The sample of patients on hypertensive treatment, who satisfied the inclusion criteria, was selected. Consecutive sampling was used, whereby the researcher selected the research subjects based on their availability and willingness to participate in a study. The researcher recruited individuals who met specific criteria one after another until the desired sample size of 200 was achieved. The sampling technique was used because it was difficult to access a large pool of potential participants, and the researcher required a specific population that was readily available. The researcher waited for hypertensive patients who went for treatment or check-ups at the health centre. Data were collected as the patients came for a period of two months. This method is reliably convenient and efficient in terms of time and cost. Participants in this study had to be 18 years or above and on hypertension medication, and they had to be willing to participate and able to give a written informed consent. Participants in this study were excluded if they were less than 18 years old, not on hypertension medication, or not willing to participate.

### Data collection tool

A semi-structured self-administered questionnaire was designed and used to collect data on the demographic characteristics and the individual-related practices of hypertensive patients. The questionnaire used in this study was developed based on previous research on factors associated with non-adherence to antihypertensive medication, research questions, and research objectives. The instrument was translated into a local language (Rukwangali) and then retranslated back into the original language (English). This practice is referred to as forward and backward translation (Tsang, Royse & Terkawi 2017). This exercise was performed to accommodate participants but fundamentally also to ensure the content validity of the instrument and/or survey, so that ‘the entire theoretical construct the questionnaire is designed to assess’ (Tsang et al. 2017:86) will measure the same in a different context. Variables included in the study were grouped into demographic characteristics, and individual-based practices associated with non-adherence to hypertensive treatment were relevant as they directly addressed the study objectives of understanding the factors influencing medication adherence among hypertensive patients. Demographic characteristics such as age, marital status, religion, education, employment status, gender, and number of children were essential for identifying socioeconomic and cultural factors that may impact adherence. Individual practices related to medication timing, discontinuation, forgetfulness, side effects, and understanding of hypertension were crucial for assessing personal behaviours and perceptions affecting treatment adherence. The predictors were demographic characteristics and individual-based practices of the participants. These variables collectively allowed the study to comprehensively evaluate the predictors of adherence. The questionnaire had 38 items, which were divided into two sections: section A had 20 items on demographic characteristics, and section B had 18 items on medication adherence and reasons for non-adherence. The variables included in the study, which were grouped into demographic characteristics and individual-based practices associated with non-adherence to hypertensive treatment, were relevant as they directly addressed the study objectives of understanding the factors influencing medication adherence among hypertensive patients. Demographic characteristics such as age, marital status, religion, education, employment status, gender, and number of children were essential for identifying socioeconomic and cultural factors that may impact adherence. Individual practices related to medication timing, discontinuation, forgetfulness, side effects, and understanding of hypertension were crucial for assessing personal behaviours and perceptions affecting treatment adherence. The predictors were the demographic characteristics and individual-based practices of the participants. These variables collectively allowed the study to comprehensively evaluate the predictors of adherence.

### Validity and reliability of the research tool

The questionnaire was pretested on four random participants to ensure the internal validity and reliability of the assessment tool in this study and to confirm whether it was able to collect data in order to meet the objectives of the study. Feedback from a pilot test did not result in any adjustments to question wording and format. Pilot study responses were not included in the main sample. The reliability test using Cronbach’s Alpha was calculated on 18 items (Section B) about perceived experiences, behaviours, and barriers (reasons) for non-adherence among hypertensive patients and had an alpha value of 0.690. The rest of the items were under Section A, which focused on factual information such as age, sex, occupation, and duration of diagnosis, which do not require consistency testing (DeVellis & Thorpe 2021; Gliem & Gliem 2003; Tavakol & Dennick 2011). For reliability, a Cronbach’s alpha greater than 0.70 is acceptable for Likert scale questions (Bujang, Omar & Baharum 2018) to determine internal consistency and stability. The instrument was also presented to the research supervisor to evaluate its content validity.

### Data collection procedure

Data were collected by using a questionnaire, which was piloted on ten participants, who did not form part of the main study. The research tool was self-administered and handed out in person to the participants. The questionnaire was paper-based; therefore, respondents wrote their answers directly on the paper, and the filled questionnaires were returned to the data collector. The completed questionnaires were kept under lock and key, and the captured data were stored on a password-protected computer.

### Data analysis

Collected data were captured into Microsoft Excel 2016 and secured with a password to ensure data integrity. At the end of the study period, the data were cleaned and checked for consistency and imported to the IBM Statistical Package for Social Sciences (SPSS) version 26.0 for analysis (Jarrar et al. 2025). Quantitative variables were effectively summarised by using frequencies and percentages. The results are presented in frequency tables with percentages (see Table 1, Table 2A, Table 2B, Table 3, Table 4 and Table 5). The Chi-square test was used as a bivariate statistical tool to identify which patient characteristics, clinical factors, behavioural practices, and perceived barriers were significantly associated with medication adherence. The binary logistic regression was used to determine the best independent predictors of medication adherence to ensure robust and reliable findings, thereby thoroughly addressing the research questions regarding demographic and behavioural factors influencing medication adherence. The dependent variable was timely medication intake, while independent variables included demographic characteristics and individual practices identified in the questionnaire. All *p*-values ≤ 0.005 were considered statistically significant (see Table 5, Table 6 and Table 7). The combination of descriptive statistics, Chi-square tests, and binary logistic regression provided a comprehensive approach to analysing the data, allowing for both a detailed description of the sample and a robust examination of the predictors of treatment adherence. This data analysis method ensured that the research questions were addressed thoroughly and that the findings were statistically sound and actionable.

**TABLE 1:** Demographic characteristics of hypertensive patients (*N* = 200).

Variable	*n*	%
**Age group (years)**		
18–39	32	16
40–59	78	39
≥ 60	90	45
**Sex**		
Male	86	43
Female	114	57
**Marital status**		
Single	48	24
Married	102	51
Divorced and/or widowed	50	25
**Education**		
No formal education	20	10
Primary	72	36
Secondary	70	35
Tertiary	38	19
**Employment status**		
Employed	64	32
Unemployed	136	68
**Family history of hypertension**		
Yes	118	59
No	82	41

**TOTAL**	**200**	**100**

**TABLE 2A:** Clinical factors and behavioural adherence practices among hypertensive patients (*N* = 200).

Variable	*n*	%
**Duration of hypertension (years)**		
< 2	56	28
2–4	62	31
≥ 5	82	41
**Current BP reading**		
Controlled (< 140/90 mmHg)	94	47
Uncontrolled (≥ 140/90 mmHg)	106	53
Medications taken (≥ 2 drugs)	128	64
Comorbidity: Diabetes mellitus	62	31
Other comorbidities (renal, etc.)	28	14

**TOTAL**	**200**	**100**

**TABLE 2B:** Clinical factors and behavioural adherence practices among hypertensive patients (*N* = 200).

Behavioural adherence practices	Always		Sometimes		Never		Total	
	*n*	%	*n*	%	*n*	%	*N*	%
Do you miss doses of your medication?	124	62	56	28	20	10	200	100
Do you forget to take your medicine?	118	59	64	32	18	9	200	100
Do you stop taking medicine when you feel well?	86	43	74	37	40	20	200	100
Do you stop when you feel worse and/or side effects?	92	46	60	30	48	24	200	100
Do you change dosage on your own?	110	55	64	32	26	13	200	100
Do you stop taking medicine when the stock runs out?	96	48	70	35	34	17	200	100

**TABLE 3:** Reasons for non-adherence to medication (*N* = 200).

Reason	*n*	%
Forgetfulness	112	56
Side effects	88	44
Financial constraints	74	37
Lack of transport	64	32
Lack of knowledge about HPT	52	26
Long waiting times at the facility	48	24
Traditional medicine use	36	18
Poor family support	30	15

HPT, hypertension.

**TABLE 4:** Health system and patient-related factors (*N* = 200).

Factor	Agree		Disagree		Total	
	*n*	%	*n*	%	*N*	%
Long waiting times discourage use	104	52	96	48	200	100
Poor communication with providers	86	43	114	57	200	100
Drug stock-outs limit adherence	92	46	108	54	200	100
Support from family helps	128	64	72	36	200	100

**TABLE 5:** Chi-square tests of association between patient characteristics and medication adherence.

Variable	χ^2^	*df*	*p*-value
Age group	4.32	3	0.229
Sex	1.14	1	0.286
Marital status	7.65	3	0.054
Educational level	9.21	3	0.027*
Employment status	6.98	2	0.031*
Family history of hypertension	2.11	1	0.146
Duration of hypertension	8.76	2	0.013*
Current BP reading	3.42	2	0.181
Comorbid diabetes mellitus	4.88	1	0.027*
Forgetfulness	11.92	1	0.001*
Lack of transport	6.02	1	0.014*
Side effects of medication	7.34	1	0.007*
Financial constraints	9.55	1	0.002*

χ^2^, Chi-squared; *df*, degrees of freedom.

* , *p*-value ≤ 0.05.

**TABLE 6:** Unadjusted logistic regression predicting medication adherence.

Variable	OR	95% CI	*p*-value
Educational level (Low vs High)	0.52	0.28–0.94	0.031*
Employment (Unemployed vs Employed)	0.47	0.23–0.88	0.018*
Duration ≥ 5 years	1.82	1.06–3.14	0.028*
Diabetes comorbidity (Yes)	0.56	0.32–0.94	0.029*
Forgetfulness	0.33	0.17–0.62	0.001*
Transport barrier	0.42	0.21–0.83	0.012*
Side effects	0.40	0.20–0.80	0.008*
Financial constraints	0.38	0.19–0.76	0.006*

CI, confidence interval; OR, odds ratio; vs, versus.

* , *p*-value ≤ 0.05.

**TABLE 7:** Adjusted logistic regression predicting medication adherence.

Variable	AOR	95% CI	*p*-value
Educational level (Low vs High)	0.61	0.29–1.05	0.073
Employment (Unemployed vs Employment)	0.52	0.24–0.97	0.041*
Duration ≥ 5 years	1.75	1.01–3.10	0.047*
Diabetes comorbidity (Yes)	0.62	0.33–1.10	0.094
Forgetfulness	0.39	0.20–0.76	0.006*
Transport barrier	0.44	0.22–0.88	0.019*
Side effects	0.42	0.21–0.89	0.011*
Financial constraints	0.40	0.21–0.82	0.009*

AOR, adjusted odds ratio; CI, confidence interval; vs, versus.

* , *p*-value ≤ 0.05.

### Ethical considerations

An application for full ethical approval was made to the University of Namibia School of Nursing Research Ethics Committee and ethics consent was received on 18 October 2021. The ethics approval number is SoN 87/2021. An application for full ethical approval was also made to the Ministry of Health and Social Services Health Ethics Committee and ethics consent was received on 27 October 2021. The ethics approval number is 17/3/3/AVN. Additionally, the gatekeepers of the hospital provided further approval and access to participants. Written informed consent was obtained from the participants before the questionnaires were administered. Participants had full rights to refuse to sign the consent or withdraw anytime from the study without penalties. Prior to signing the consent, participants were informed about the purpose, objective, data collection procedure, advantages, and disadvantages of the study. During this study, codes were assigned and used on behalf of participants’ real names to ensure anonymity, and the data collected were solely used for the purpose of the study. Participants had complete privacy and confidentiality of the information they were going to give throughout the interview. Moreover, the completed questionnaires were kept under lock and key to ensure confidentiality, and only the researcher and supervisor had access to the questionnaires. All eligible participants were given equal information about the study and an equal chance to voluntarily participate therein.

## Results

### Demographic characteristics

The demographic characteristics in Table 1 show that the majority of patients were older adults, with almost half of them being 60 years and above (90 out of 200; 45%). Women made up more than half of the sample (114; 57%), while men represented a smaller share (86; 43%). More than half of the participants were married (102; 51%), while 25% (50) were divorced or widowed, and 24% (48) were single. In terms of education, 20 patients (10%) had no formal schooling, 72 (36%) had only primary education, 70 (35%) completed secondary school, and 38 (19%) had tertiary qualifications. Employment was also skewed, with more than two-thirds unemployed (136; 68%) and only 64 (32%) reporting employment, while 59% reported a family history of hypertension. Importantly, both lower education (χ^2^ = 9.21, *p* = 0.027) and unemployment (χ^2^ = 6.98, *p* = 0.031) were significantly associated with poor adherence (Table 6), suggesting that limited literacy and financial hardship can undermine consistent medication-taking.

Table 2A and Table 2B highlights the clinical and behavioural profiles. A large number of patients had been living with hypertension for at least 5 years (82; 41%), while 62 (31%) had lived with it for 2–4 years and 56 (28%) for less than 2 years. Only 94 patients (47%) had controlled BP at the time of the study, while the rest (106; 53%) were uncontrolled. Nearly two-thirds (128; 64%) were taking two or more antihypertensive drugs, while 62 patients (31%) had diabetes as a comorbidity, and another 28 (14%) reported other conditions such as renal problems. Duration of hypertension was significantly associated with adherence (χ^2^ = 8.76, *p* = 0.013) (Table 6), and logistic regression showed that patients with hypertension for ≥ 5 years had higher odds of adherence (adjusted odds ratio [AOR] = 1.75, 95% confidence interval [CI]: 1.01–3.10, *p* = 0.047) as shown in Table 7. Diabetes, on the other hand, was associated with lower adherence in the unadjusted model (odds ratio [OR] = 0.56, 95% CI: 0.32–0.94, *p* = 0.029) (Table 7), although this was not significant in the adjusted model.

Table 2A and Table 2B further reveals worrying trends in the behavioural practices. More than half of the patients admitted that they ‘always’ miss doses (124; 62%) or forget to take their medicine (118; 59%). A considerable proportion reported stopping their medication when they felt well (86; 43%) or when they experienced side effects (92; 46%). Over half (110; 55%) even said that they changed their dosage on their own, while almost half (96; 48%) stopped treatment when their medicine ran out. The Chi-square test in Table 6 confirmed that forgetfulness (χ^2^ = 11.92, *p* = 0.001) was strongly linked to non-adherence, and logistic regression in Table 7 showed that patients who reported forgetfulness were far less likely to adhere (AOR = 0.39, 95% CI: 0.20–0.76, *p* = 0.006). This finding makes forgetfulness the single strongest behavioural barrier to adherence in this study.

Table 3 explains in more detail the reasons for non-adherence. The leading cause was forgetfulness, reported by 112 patients (56%). Side effects were the second-most common reason (88; 44%), followed by financial constraints (74; 37%), lack of transport (64; 32%), and lack of knowledge about hypertension (52; 26%). Nearly a quarter (48; 24%) mentioned long waiting times, while 36 patients (18%) turned to traditional medicine, and 30 (15%) reported poor family support. Financial constraints were statistically significant (χ^2^ = 9.55, *p* = 0.002), and patients facing financial barriers had much lower odds of adherence (AOR = 0.40, 95% CI: 0.21–0.82, *p* = 0.009) (Table 7). Similarly, side effects (χ^2^ = 7.34, *p* = 0.007) were a major deterrent (Table 6), with patients who reported side effects showing less than half the odds of adhering to medication (AOR = 0.42, 95% CI: 0.21–0.89, *p* = 0.011) (Table 7). Transport barriers also mattered (χ^2^ = 6.02, *p* = 0.014), cutting adherence odds by more than half (AOR = 0.44, 95% CI: 0.22–0.88, *p* = 0.019). These findings reinforce how practical obstacles and the unpleasant realities of treatment can lead patients to default.

Health system and patient-related issues in Table 4 confirm these struggles. Just over half of the patients (104; 52%) said that long waiting times discouraged them from using services, while 92 (46%) reported that drug stock-outs limited adherence. Almost as many (86; 43%) felt that communication with health providers was poor. Encouragingly, however, 128 patients (64%) felt that family support helped them take their medicine. This finding demonstrates that the health system poses real access barriers, but social support can play an important protective role in adherence.

The statistical tests in Table 5, Table 6, and Table 7 put all of these factors into perspective. Among demographic and socioeconomic factors, education, employment, and marital status showed important associations, although only employment remained significant after adjustment (AOR = 0.52, *p* = 0.041). Among clinical factors, longer duration of hypertension was associated with better adherence, while diabetes comorbidity weakened adherence in the unadjusted analysis. Among behavioural and patient-reported factors, the most powerful predictors were forgetfulness (AOR = 0.39, *p* = 0.006), side effects (AOR = 0.42, *p* = 0.011), financial constraints (AOR = 0.40, *p* = 0.009), and transport barriers (AOR = 0.44, *p* = 0.019).

When the findings are considered together, the study paints a picture of patients who are willing to treat their condition but are hindered by low socioeconomic status, daily struggles with remembering and tolerating medication, and systemic barriers such as transport and long queues at facilities. Employment and longer experience with hypertension increased adherence, showing that stability and adaptation make a difference. By contrast, forgetfulness, side effects, financial strain, and transport difficulties cut adherence dramatically, sometimes halving the odds. The results emphasise that in order to improve adherence, a multifaceted approach is required. This approach includes strengthened patient education, designed reminder systems to counter forgetfulness, counselling offered and medication adjustments to address side effects, and reduced financial and logistical barriers that patients face. Family support also emerges as a powerful protective factor, which suggests that when households and communities are involved, treatment adherence could be made more sustainable.

## Discussion

This study examined demographic, clinical, behavioural, and health system factors associated with medication adherence among hypertensive patients at a regional hospital in the Kavango East region, Namibia. The findings show that adherence in this setting is shaped by a mix of social, clinical, behavioural, and health system realities. Socioeconomic vulnerability emerged clearly: most participants were unemployed (136; 68%), and a sizable portion had only primary or no formal education (92; 46%). In our adjusted analysis, unemployment was associated with about half the odds of adherence compared with being employed (AOR = 0.52, 95% CI: 0.24–0.97, *p* = 0.041). This observation reinforces patterns seen elsewhere in sub-Saharan Africa where poverty and unstable livelihoods make steady treatment more difficult, both because of the direct costs of care and because uncertain daily life disrupts routines (Nakwafila et al. 2022; Ogungbe et al. 2021). Addressing socioeconomic constraints is therefore not peripheral but central to any adherence strategy in Kavango East.

Clinical context is complex but instructive. Patients with longer experience of hypertension (≥ 5 years) were more likely to adhere (AOR = 1.75, 95% CI: 1.01–3.10, *p* = 0.047), suggesting that habituation and experience often build routines that support medication-taking. At the same time, comorbidity with diabetes undermined adherence in unadjusted analysis (OR = 0.56, 95% CI: 0.32–0.94, *p* = 0.029) and trended towards reduced adherence after adjustment (AOR = 0.62, *p* = 0.094). Diabetes, on the other hand, was associated with lower adherence in the unadjusted model (OR = 0.56, 95% CI: 0.32–0.94, *p* = 0.029) (Table 7), although this was not significant in the adjusted model. This pattern suggests that patients who have lived with hypertension for longer may become more accustomed to the routine of taking medication, while those managing multiple conditions like diabetes may struggle with the complexity of treatment.

Managing multiple chronic conditions creates clinical complexity; more pills imply more possible drug interactions and side effects, which can overwhelm patients and reduce adherence unless care is coordinated. These findings match broader literature showing that patients with multiple chronic conditions often face greater difficulties in achieving consistent medication use unless services are coordinated and regimens simplified (Dean et al. 2024). The implications are clear: clinical care should prioritise treatment simplification (for example, use of fixed-dose combinations where appropriate) and integrated management of comorbidities to reduce the practical complexity patients face.

Behavioural drivers were particularly prominent. More than half the sample admitted to routinely missing doses (124; 62%) and forgetting medication (118; 59%). Forgetfulness had a large, independent negative effect on adherence (AOR = 0.39, 95% CI: 0.20–0.76, *p* = 0.006), making it the strongest single behavioural predictor in our models. This effect mirrors evidence from multiple contexts showing that forgetfulness is among the most common and most addressable causes of non-adherence (Belete et al. 2024; Tam et al. 2021). Side effects were also a frequent reason to stop treatment (88; 44%) and independently reduced adherence (AOR = 0.42, 95% CI: 0.21–0.89, *p* = 0.011). Trials and meta-analyses indicate that reducing pill burden with fixed-dose combination pills can improve adherence, in part because fewer tablets and more predictable schedules tend to reduce both missed doses and discontinuation from side effects (Wei et al. 2023). Clinical teams must therefore routinely elicit side effects and be ready to modify regimens or counsel patients so that tolerability does not become a reason to abandon therapy.

The health system and logistical barriers reinforced individual challenges. Financial constraints were reported by 74 patients (37%) and were associated with much lower odds of adherence (AOR = 0.40, 95% CI: 0.21–0.82, *p* = 0.009). Transport difficulties affected 64 patients (32%) and also halved the odds of adherence (AOR = 0.44, 95% CI: 0.22–0.88, *p* = 0.019). Nearly half the sample (92; 46%) identified drug stock-outs as a limiting factor, and 104 patients (52%) said long waiting times discouraged use. These structural obstacles are consistent with qualitative and implementation studies from low- and middle-income settings showing that physical access, inconsistent supply, and opportunity costs (time away from work and/or travel costs) are major drivers of poor chronic disease control (Mbuthia, Magutah & Pellowski 2022; Nguyen & Nyame 2024). Simply advising patients to ‘take your pills’ is ineffective if medicines are unavailable or a clinic visit costs a day’s wages.

Framing these findings with behaviour-change theory helps translate them into realistic interventions. The COM-B model (capability, opportunity, motivation → behaviour) explains several of our observations (Michie, Van Stralen & West 2011). Many patients lacked the psychological or organisational capability to remember (high rates of forgetfulness), the physical opportunity was limited by transport and stock-outs, and financial strain lowered motivation to prioritise medicines over other needs. The Health Belief Model also explains some choices: perceived barriers (cost, side effects, and/or waiting times) appeared to outweigh perceived benefits for many, so the net result was non-adherence (Rosenstock 1974). Using these frameworks together suggests that interventions must simultaneously build capability (education and/or reminders), expand opportunity (community drug distribution, reliable supply, and/or reduced travel), and strengthen motivation (counselling, family engagement, and/or incentives).

Fortunately, a body of contemporary evidence supports feasible interventions that map well to the problems identified here. Short message service and mobile reminders have a strong evidence base for improving adherence, and, in some trials, BP outcomes; one meta-analysis and several recent reviews indicate that one-way or two-way text messaging can be effective when implemented with local tailoring and integration into care (Belete et al. 2024; Tam et al. 2021; Zhai et al. 2020). Community health worker (CHW) programmes and task-shifting have improved linkage, adherence, and BP in multiple LMIC studies, and they also address transport and access problems by bringing care closer to patients (Mbuthia et al. 2022; Nguyen & Nyame 2024). Simplifying drug regimens using fixed-dose combination pills reduces pill burden and improves adherence and BP control; meta-analyses show a consistent benefit of combination pills over separate-drug regimens (Wei et al. 2023). Financial interventions, such as subsidised medicines, conditional cash transfers, or broader social protection schemes, have also been shown to improve adherence and should be considered where cost is a barrier (Petry et al. 2012; recent reviews on financial assistance for medicines).

Bringing these strands together implies a multifaceted package for Kavango East. Routine screening for adherence problems should include simple questions about forgetfulness, side effects, and practical barriers; this inclusion is justified by our finding that forgetfulness (118; 59%) and side effects (88; 44%) were common and strongly predictive of non-adherence. Low-cost reminder strategies such as short message service, voice reminders for those with limited literacy, or family-supported pill boxes could directly target forgetfulness and are supported by recent evidence (Belete et al. 2024; Tam et al. 2021). Clinical protocols should prioritise side-effect management and consider fixed-dose combinations to lower pill burden where clinically appropriate (Wei et al. 2023). Health system responses should include multi-month dispensing, decentralised drug pick-up points, or CHW-led distribution to reduce transport and waiting-time burdens and strengthened supply-chain management to avoid stock-outs (Mbuthia et al. 2022; Nguyen & Nyame 2024). Finally, social protection and targeted subsidies for those who are unemployed (136; 68%) should be explored because financial constraints (74; 37%) had an independent negative effect on adherence. These recommendations reflect both our data and our interventions with evidence of effectiveness in similar low-resource settings (Hung et al. 2021; Ogungbe et al. 2021).

### Limitations

The study was cross-sectional, so causal direction cannot be proven; for example, people who are more adherent may be more likely to remain employed, or conversely, unemployment may worsen adherence. Self-reported adherence and reasons for non-adherence are vulnerable to recall and social-desirability biases; nonetheless, the convergence of self-report with objective clinical markers such as uncontrolled BP (106; 53%) supports the overall validity of the signal. The sample is drawn from a single regional hospital, and patterns may differ in urban centres or other Namibian regions; however, the similarity between our findings and other Namibia studies (Nakwafila et al. 2022) and LMIC reviews suggests that many of the determinants identified are not unique to Kavango East. Future research should test bundled interventions (for example, reminders plus decentralised dispensing plus CHW follow-up) in implementation trials that measure both adherence and clinical outcomes such as BP control and cardiovascular events.

## Conclusion

Adherence among hypertensive patients in this Namibian regional hospital is affected by a combination of forgetfulness and treatment tolerability, combined with socioeconomic hardship and structural access problems. Interventions that combine patient-level supports (reminders, counselling, and simplified regimens), community approaches (CHW distribution and follow-up), and system changes (reliable drug supply, multi-month dispensing, and financial protection) are likely to be the most effective. These measures align with current global guidance and evidence, and they respond directly to the lived realities reported by patients in this study. Future implementation work should pilot and evaluate a bundled intervention that addresses capability, opportunity, and motivation together, measuring both adherence and downstream outcomes such as BP control and cardiovascular events.
